# Deuterium-Depleted Water as Adjuvant Therapeutic Agent for Treatment of Diet-Induced Obesity in Rats

**DOI:** 10.3390/molecules25010023

**Published:** 2019-12-19

**Authors:** Tetiana Halenova, Igor Zlatskiy, Anton Syroeshkin, Tatiana Maximova, Tatiana Pleteneva

**Affiliations:** 1 Taras Shevchenko National University of Kyiv, ESC “Institute of Biology and Medicine”, 64 Volodymyrska Str., 01601 Kyiv, Ukraine; galenovatanya@gmail.com; 2 Peoples Friendship University of Russia (RUDN University), 6 Miklukho-Maklaya St, Moscow 117198, Russian; livmatter@mail.ru (A.S.); maximtat@mail.ru (T.M.); tvplet@mail.ru (T.P.); 3 State Institute of Genetic and Regenerative Medicine NAMS of Ukraine, 67 Vyshgorodska Str., 04114 Kyiv, Ukraine

**Keywords:** deuterium-depleted water, diet-induced obesity, body weight index, oxidative stress, cytokines

## Abstract

In this study, we present the potential application of deuterium-depleted water (DDW) for the prevention and adjuvant treatment of obesity in rats. We tested the hypothesis that DDW can alleviate diet-induced obesity (DIO) and its associated metabolic impairments. Rats fed a high-fat diet had an increased body weight index (BWI), glucose concentration, and level of certain proinflammatory cytokines; decreased levels of insulin in the serum; decreased tryptophan and serotonin in the brain, and a decreased concentration of some heavy metals in the liver. Drinking DDW at a concentration of 10 ppm deuterium/protium (D/H) *ad libitum* for 3 weeks restored the BWI, glucose (serum), tryptophan (brain), and serotonin (brain) levels and concentration of Zn in the liver in the DIO animals to those of the controls. The levels of proinflammatory cytokines (IL-1β, IL-6, IFNγ) and anti-inflammatory TNFα were decreased in DIO rats, while anti-inflammatory cytokine (IL-4, IL-10) levels remained at the control levels, which is indicative of a pathophysiological syndrome. In contrast, in groups of rats treated with DDW, a significant increase in anti-inflammatory (IL-4, IL-10) and proinflammatory cytokines (IFNγ) was observed. This finding indicates a reduction in systemic inflammation in obese animals treated with DDW. Similarly, the high-fat diet caused an increased level of oxidative stress products, which was accompanied by decreased activity of both superoxide dismutase and catalase, whereas the administration of DDW decreased the level of oxidative stress and enhanced antioxidant enzyme activities.

## 1. Introduction

Obesity is one of the major problems of global significance [1]. This pathological condition is strongly associated with multiple comorbidities, including different metabolic complications that can affect almost every human organ system. Obesity is a major risk factor for insulin resistance [2], diabetes [3], cardiovascular disease [4] and certain forms of cancer [5]. Excessive fat accumulation in the body can also lead to pathological changes in respiratory and musculoskeletal physiology [6]. Currently, there is a lack of effective therapeutic approaches for the treatment of obesity and prevention of its related complications. Over recent decades, there has been increased interest in the use of dietary supplements and pharmaceuticals for obesity treatment. However, it should be noted that these strategies are usually associated with frequent cases of adverse gastrointestinal events. Therefore, the development of new, safer and more effective pharmaceutical approaches for obesity treatment is essential.

Natural water is a mixture of molecules containing stable isotopes ^16^O, ^17^O, ^18^O, ^1^H, and ^2^H. The ratio of deuterium atoms to protium (D/H) in fresh and marine water is 132–156 µg/g (ppm) [7,8]. Since the mass of protium H is half of the mass of deuterium D, effects arising from the replacement of isotopes influence biological systems; for example, the rates of chemical reactions between substances containing these isotopes may differ by a factor of 5–10 [9,10,11].

Numerous publications over the last 20 years have been devoted to the study of the role of deuterium in natural (150 ppm) or reduced content in the human body [12]. It has been shown that deuterium-depleted water (DDW) exhibits antidotal properties with individual and combined effects on the dosage of pharmaceutical substances and auxiliary substances [13,14,15,16]. The mechanisms of such influence are due to the structure, physicochemical properties of DDW [17] and changes in ligand-receptor interactions in biological objects of different hierarchical levels [18]. DDW increases the rate of photosynthesis and promotes the growth of plants and aquatic animals [19,20]. In Europe, the USA, Japan, China and Russia, DDW is used for prophylactic and therapeutic purposes [21,22]. Currently, DDW is using as an adjuvant in cancer treatment [22]. Limited data have shown that deuterium in aqueous solutions can stimulate or inhibit metabolic processes in living systems [23,24,25,26,27]. As mentioned above, DDW has a number of unexpected biological properties, including antitumor [28], antidotal [29] and metabolic effects [30]. However, whether deuterium is involved in the regulation of biological properties of in-vivo models has not been fully characterized [26,27]. Differences in the D/H ratio is manifested in the form of kinetic isotope effects, characterized by a change in the biotransformation and excretion rates of the drugs [31,32,33]. Because of its characteristics, DDW has attracted much interest in the context of obesity. The positive effects of using DDW make it a promising adjuvant.

Therefore, the aim of this study was to determine the effects of DDW as a potential adjuvant therapeutic agent for the treatment of obesity and obviation of its metabolic complications within in-vivo model on rats.

## 2. Results

### 2.1. Somatometric and Nutritional Parameters in Control and DIO Rats that Consumed either MilliQ Water or DDW

**Figure 1:**
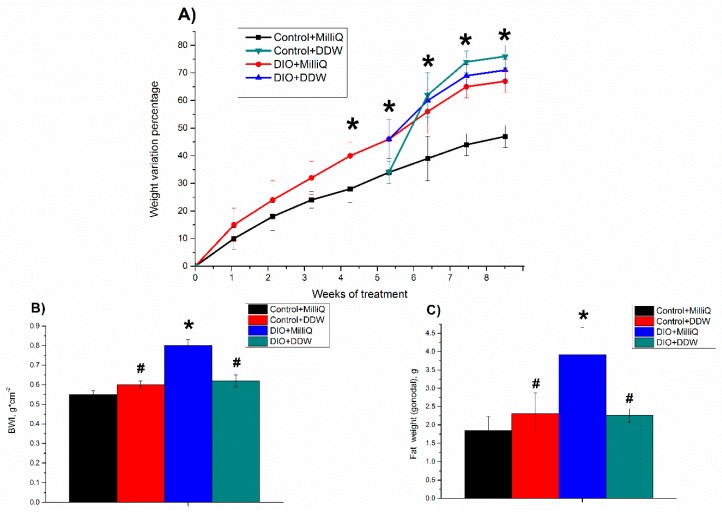
Time course of weekly percentage variation in body weight: (**A**); body weight index (BWI) (**B**); and gonadal fat weight (**C**), at the end of the 8th week in experimental groups of rats. The 0% value indicates the basal body weight of each rat. Values are expressed as the mean ± SD (*n* = 10); * *p* < 0.05 significantly different from the Control + MilliQ group; # *p* < 0.05 significantly different from the diet-induced obesity (DIO) + MilliQ group.

The weight variation percentage with respect to the initial weight of all experimental groups of animals during the 8 weeks is shown in Figure 1A. During the first 5 weeks of the experiment, the body weight of the Control + MilliQ rats was elevated by 30% from the beginning of the monitoring period. The body weight gain of rats fed a high-fat diet was significantly higher than that of the control. The initial weight of the DIO + MilliQ rats increased by 45% for the first 5 weeks of the experiment.

On the 35th day, the control and DIO rats were divided into two subgroups. Rats of the first subgroup continued to drink MilliQ water (H/D = 150 ppm), while the rats of the second subgroup, for the next three weeks, drank DDW (H/D = 10 ppm).

The final body weight of the animals in the Control + MilliQ group was 45% greater than the initial body weight, while the final body weight of the animals in the Control + DDW group was 75% greater than the initial body weight.

After 8 weeks of consumption, the body weight of the high-fat diet rats in the DIO + MilliQ group was increased by 70% compared with the initial weight, and the rats in the DIO + DDW group showed a similar weight gain to the rats in the DIO + MilliQ group.

Our results showed an increase in BWI in the DIO + MilliQ rats compared to the Control + MilliQ animals, which indicates the development of obesity (Figure 1B). Drinking of DDW by the control animals had no effect on the BWI compared to that of the Control + MilliQ animals. Drinking of DDW by the DIO rats resulted in the decrease in this parameter by 25% (*p* < 0.05) compared to that of the DIO + MilliQ rats (Figure 1B).

Our results showed a more than twofold increase in gonadal fat weight in the DIO + MilliQ rats compared to the Control + MilliQ animals, which indicates the development of obesity. Drinking of DDW by the control animals had no effect on gonadal fat accumulation compared to the drinking of MilliQ water. The DIO + DDW group showed a 1.7-fold decrease (*p* < 0.05) in gonadal fat weight compared to the DIO + MilliQ group (Figure 1C).

**Figure 2:**
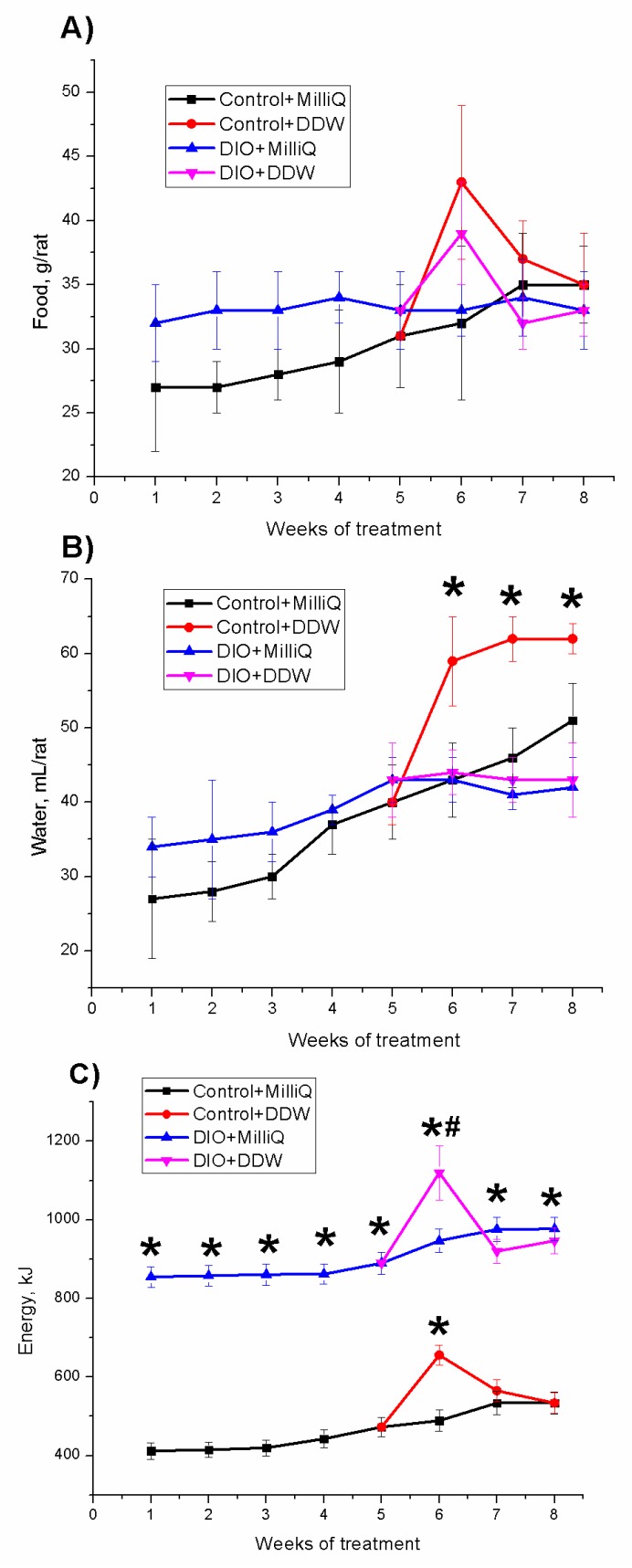
Mean daily food intake (**A**), water intake (**B**), and energy intake (**C**) of control and DIO rats that consumed MilliQ water or DDW for 3 weeks of the DIO experiment. Values are expressed as the mean ± SD (*n* = 10); * *p* < 0.05 significantly different from the Control + MilliQ group; # *p* < 0.05 significantly different from the DIO + MilliQ group.

Mean values of daily food and water intake for all experimental groups of animals over 8 weeks are shown in Figure 2A,B, respectively.

For the first five weeks, we observed a linear increase in the consumption of food by rats in the Control + MilliQ and DIO + MilliQ groups (in the DIO + MilliQ group, there was no statistically significant increase compared to the Control + MilliQ group) (Figure 2A). When rats in both the control and DIO groups started to drink DDW, their food consumption increased. At the end of the first week of DDW drinking, the food consumption was increased by 40% in the control and DIO rats compared to the corresponding MilliQ rats. However, at the end of the experiment in both DDW groups of control and DIO rats, food consumption was decreased to the corresponding initial level. This observation can be explained by the adaptation of the rats to the change in the ratio of D/H in drinking water.

As with the consumption of food, for the first five weeks we observed a linear increase in the consumption of water by rats in the Control + MilliQ and DIO + MilliQ groups. No significant difference was observed in water consumption by the rats in the DIO + MilliQ and Control + MilliQ groups for the first five weeks (Figure 2B). When rats in both the control and DIO groups began to drink DDW, we observed different changes in the DIO and control groups. In the DIO + DDW group, a nonsignificant decrease in water consumption was observed compared to the DIO + MilliQ group. However, in the Control + DDW group, a significant increase (by 50%) in water consumption was observed compared to the Control + MilliQ group.

Because of the different feeding patterns, the groups of control and high-fat diet rats had different energy intake statuses, as shown in Figure 2C. During the experiment, the Control + MilliQ rats consumed significantly reduced amounts of energy (Figure 2C). The energy consumption in the Control + MilliQ group was 2.2-fold lower than that in the DIO + MilliQ group. In both groups of high-fat diet rats DIO + MilliQ and DIO + DDW, the average energy consumption per rat during the entire monitoring period did not change significantly (Figure 2C), except for an increase in energy intake in the first week after the introduction of DDW. The Control + DDW group showed no statistically significant increase in energy consumption compared to the Control + MilliQ group, except for the increase in energy intake in the first week after the introduction of DDW.

### 2.2. Biochemical Parameters at the End of the Experiment in Control and DIO Rats that Consumed either MilliQ Water or DDW

Obesity is a pathological state closely related to the impairment of glucose metabolism and insulin resistance development [2,34,35]. Indeed, as depicted in Table 1, the high-fat diet led to a 1.4-fold increase in blood glucose concentration (*p* < 0.05) as well as a 2.5-fold decrease in serum insulin level (*p* < 0.05) compared to the same parameters in the control rats. Such changes in the studied parameters demonstrate abnormalities in glucose metabolism. The development of a prediabetic state seems to have occurred in the DIO + MilliQ rats.

The consumption of DDW by DIO rats led to a significant reversion of the effects of the high-fat diet on the glucose concentration but not the insulin level. Thus, the concentrations of glucose in DIO + DDW rats were close to those of the control animals (*p* > 0.05), whereas the concentration of serum insulin in this group decreased 2.6 times (*p* < 0.05) compared to that of the Control + MilliQ group. The concentrations of glucose and serum insulin in the Control + DDW rats were the same as those in the Control + MilliQ rats (*p* > 0.05).

### 2.3. Antioxidant Enzyme Activity in Serum at the End of the Experiment in Control and DIO Rats that Consumed either MilliQ Water or DDW

Our activation results of two antioxidant enzymes, SOD and catalase, in the serum of DIO rats are consistent with previous observations [35,36].

A significant decrease in the activities of the studied antioxidant enzymes in the DIO + MilliQ group in comparison to the Control + MilliQ group was observed (Table 1): SOD activity was reduced as much as 5-fold (*p* < 0.05), whereas catalase activity was decreased more than 2.5-fold (*p* < 0.05). The consumption of DDW by DIO rats increased the activities of the antioxidant enzymes compared to the consumption of MilliQ water. The SOD activity was elevated 2.3-fold (*p* < 0.05), whereas the catalase activity was increased almost 2.5-fold (*p* < 0.05) compared to that of the DIO + MilliQ rats. Nevertheless, the activity of SOD in the DIO + DDW group remained significantly decreased by a factor of 2.5 (*p* < 0.05) compared to that of the Сontrol + MilliQ group. The catalase activity in the DIO + DDW group was the same as that in the Сontrol + MilliQ group. It should be noted that the activity of catalase in Control + DDW rats was the same (*p* > 0.05) as that in the Сontrol + MilliQ rats, whereas the SOD activity in the Control + DDW group was decreased 1.7-fold (*p* < 0.05) compared to that in the Сontrol + MilliQ group (Table 1).

### 2.4. Serum Cytokine Profile at the End of the Experiment in Control and DIO Rats that Consumed either MilliQ Water or DDW

**Figure 3:**
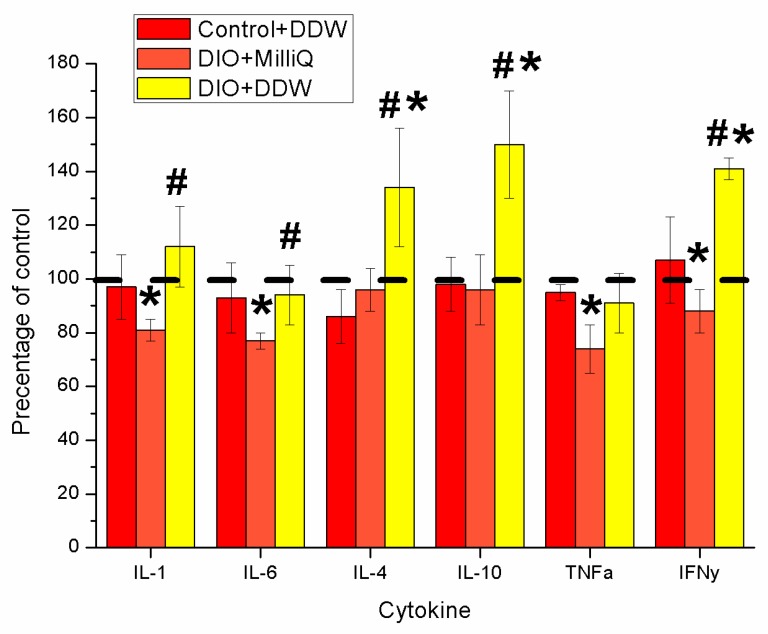
The end-of-experiment serum cytokine profile of control and DIO rats that consumed MilliQ water or DDW. The levels of the studied cytokines are expressed as a percentage of their level relative to those of the Control + MilliQ group, which were set as 100%. * *p* < 0.05 significantly different from the Control + MilliQ; # *p* < 0.05 significantly different from the DIO + MilliQ.

Obesity causes alterations not only in lipid storage but also in the composition of adipose-resident immune cell populations, leading to changes in cytokine and hormone expression [35,37,38,39,40]. Therefore, we studied whether DDW affects the levels of pro- and anti-inflammatory cytokines (Figure 3).

Figure 3 shows the results of our study of various cytokine levels in the serum of rats fed a high-fat diet with or without DDW intervention compared to the Control + MilliQ rats. The levels of the studied cytokines in the Control + MilliQ group of animals were set at 100%, and changes in their concentrations are given as the percentage change relative to the Control + MilliQ group.

In the group of DIO + MilliQ animals, we observed a decrease in proinflammatory cytokines: the levels of IL-1β and IL-6 were decreased by more than 20% (*p* < 0.05), and IFNγ was reduced by 12% (*p* < 0.05) compared with the corresponding Control + MilliQ values (Figure 3). We did not observe changes (*p* > 0.05) in anti-inflammatory cytokine (IL-10, IL-4) levels in the serum of DIO + MilliQ rats, whereas the level of serum TNFα in DIO animals was decreased by 28% (*p* < 0.05) compared to that of Control + MilliQ rats (Figure 3).

In DIO animals, after 3 weeks of DDW consumption, we observed significantly increased levels of the anti-inflammatory cytokines IL-4 and IL-10, by more than 50% (*p* < 0.05) and more than 35% (*p* < 0.05), respectively, compared with those of Control + MilliQ animals. The level of IFNγ was also increased in the serum of DIO + DDW animals by 40% (*p* < 0.05) compared with the Control + MilliQ levels (Figure 3). Concentrations of other cytokines in DIO + DDW rats were the same as those in the Control + MilliQ rats. The concentrations of cytokines in the Control + DDW group of rats were the same as those in the control animals consuming MilliQ water.

### 2.5. End of Experiment Levels of Tryptophan and Serotonin in the Brains of Control and DIO Rats that had Consumed MilliQ Water or DDW

The metabolism of brain serotonin, one of the key factors in feeling satiety, can be affected by obesity [41,42]. Indeed, we found a significant decrease in the levels of serotonin and its metabolic precursor tryptophan in the brains of DIO rats (2 and 1.9 times, respectively, *p* < 0.05) compared with those in the brains of the Control + MilliQ rats (Table 1).

In the Control + DDW and DIO + DDW groups, the levels of serotonin and tryptophan did not differ significantly from those of the Control + MilliQ group (*p* > 0.05). These results may indicate a positive effect of DDW on serotonin metabolism in the brains of DIO rats.

### 2.6. End of Experiment Levels of Certain Heavy Metals in the Livers of Control and DIO Rats that had Consumed either MilliQ Water or DDW

Zn, Mn and Cu are involved in the synthesis of various hormones associated with obesity [43,44]. We investigated the concentration of these heavy metals in the livers of DIO rats that consumed water with different deuterium levels (Table 1). In the DIO + MilliQ group, a significant (*p* < 0.05) decrease in Zn and Mn content was noted in comparison with the Control + MilliQ group. There were no changes (*p* > 0.05) in the concentrations of all studied metals in the Control + DDW and DIO + DDW groups in comparison with the Control + MilliQ group.

## 3. Discussion

The protective properties of DDW have been confirmed by toxicological studies, from which it follows that DDW, due to its transport properties, effectively removes toxins and metabolic products from the body [18,45,46]. The change in the D/H ratio manifests in the form of kinetic isotopic effects characterized by a change in the rate of absorption, distribution, biotransformation and excretion of medications [26,47,48].

It has previously been shown that using DDW accelerates metabolism, intracellular processes associated with the inhibition of oxidative stress development under various pathological conditions in vitro [27,49,50,51]. Since oxidative stress is a major pathophysiological factor in the development of obesity and the progression of obesity-related complications, we hypothesized that DDW may also alleviate these conditions. To test this hypothesis, we used a rat DIO model [34,37,52]. To determine the effect of DDW on nutritional and metabolic parameters, rats were fed either a normal diet or a high-fat diet for 56 days and had *ad libitum* access to DDW (instead of MilliQ water) from the 35th day from the start of the experiment.

According to our results (Figure 1A,B), at the end of the 56-day experiment, DIO + MilliQ rats were significantly heavier and had higher BWIs than standard chow-fed rats (Control + MilliQ). Moreover, the increased accumulation of gonadal fat demonstrated additional development of obesity in rats of the DIO + MilliQ group (Figure 1C).

The data obtained in this study indicates that, although rats in the DIO + DDW group after three weeks of DDW use had the same weight as the rats in the DIO + MilliQ group they had a lower BWIs (Figure 1A,B). Moreover, we found that the weight gain in the first few weeks of DDW use was accompanied by increased food and water intake (Figure 2A,B), which, in addition to the nonsignificant weight loss, could indicate an increase in the metabolic processes intensity of the fat rats consuming DDW. The Control + DDW group confirmed this hypothesis, and there was a significant increase in body weight along with a low BWI compared to that of the Control + MilliQ group. Based on our results, we conclude that DDW alleviates obesity caused by a high-fat diet. This finding confirms the expenditure of energy, which compared with the DIO + MilliQ group, increased in the first weeks and then decreased in the DIO + DDW group (Figure 2).

Obesity is closely associated with multiple metabolic alterations that are risk factors for glucose homeostasis abnormalities [2,35,37,52,53]. Decrease in the serum insulin level was observed in the DIO + MilliQ group of animals (Table 1). In addition, we found that long-term consumption of high-calorie food also resulted in enhanced fasting blood glucose concentrations in experimental rats. It has been proven that the degree of adiposity and the site of body fat localization influences insulin metabolism [35,37,54,55].

Reduction in the studied metabolic parameters was demonstrated after DDW consumption. The present findings indicated a decrease in both blood sugar and serum insulin (Table 1) levels after 3 weeks of DDW consumption by DIO rats. We hypothesized that the normalization of these parameters in DIO + DDW animals may be due to the positive effect of DDW on hepatic and muscle tissue functions caused by its remarkable antioxidant capacity [47,56,57]. This hypothesis is confirmed by present findings showing that DDW can overcome oxidative stress development by increasing metabolism [18,31,58].

The mechanism for the occurrence of the insulin resistance due to obesity conditions has not been well studied. The low level of insulin in the DIO + MilliQ group may also correlate with low levels of Zn and Mn in the rat liver (Table 1). Zinc is involved in the synthesis of various anabolic hormones in the body, including insulin, testosterone and growth hormone [59,60]. A lack of Zn in the body leads to a number of disorders associated with obesity [61]. In addition, changes in zinc levels in the body are a marker of various inflammatory processes, including obesity [62,63]. In our experiment, we observed a recovery of Zn levels in the DIO + DDW group to those of the Control + MilliQ group.

We found that long-term DDW consumption by DIO rats normalized the activities of the antioxidant enzymes SOD and CAT. This observation is consistent with previous data regarding the protective effect of DDW on the antioxidant system of the body in various pathologies [18,20,26,50,64,65].

Our present findings showed a decrease in the serum levels of the proinflammatory cytokines IFNγ, IL-1β, and IL-6 and a decrease in the level of anti-inflammatory TNFα in the DIO + MilliQ group (Figure 3). This result could be due to the enhanced release of proinflammatory cytokines from adipose tissue under obese conditions in the initial stages and then a sharp decrease in the acute phase of the disease, which has been proven by previous studies [66,67,68,69,70,71]. Thus, lower levels of proinflammatory cytokines are associated with the fact that the rats in the DIO + MilliQ group are unable to address the consequences of obesity.

Importantly, relative to the levels of controls, elevated serum levels of the anti-inflammatory cytokines IL-4 and IL-10 as well as proinflammatory IFNγ were found in DIO rats treated with DDW for three weeks (Figure 3). Our findings may indicate a reduction in systemic inflammation in obese animals treated with DDW. Perhaps this result is due to the ability of deuterium to slow down or speed up the reaction rate of intracellular processes include decreased systemic oxidative stress resulting in restoration of the normal functioning of the liver, adipose and muscle tissues and/or an influence of DDW on the balance between the production of pro- and anti-inflammatory cytokines [29,30]. DDW has previously been shown to have chirality capabilities mediated, partially or completely by alterations in metabolic pathways in live systems [18].

We observed that DIO leads to decrease in the levels of tryptophan and serotonin in the brain (Table 1). In obesity, reduced serotonin levels can cause disruption to the effect of satiety and the development of hyperphagia [72,73,74,75,76]. We found that the DDW-normalized serotonin and tryptophan levels in the brain impaired by DIO (Table 1).

The effect of DDW has both theoretical and important practical applications, which apply to the use of DDW as an adjuvant, where the possible contribution of deuterium to its biological activity should be considered. The result of the possible changes induced by DDW use manifest in effects [18] characterized by a change in the rate of absorption, distribution, biotransformation, and excretion of medications. The development of methodological approaches to treating obesity with DDW based on changes in drug properties will improve the pharmaceutical analysis and optimization of medication dosages, reducing the toxic load on the body. Therefore, the focus of future research should be revealing the mechanism by which DDW suppresses the chronic inflammation involved in a range of diseases in addition to obesity. Our data may not be related to the DDW factor until an exact mechanism of action has been described. However, the effect of deuterium on the change of biochemical parameters values is of interest to the scientific community [13,14,15,16,25,32,47,50,56,57,64,77] et al. Therefore, our data will contribute to the study of mechanisms of action in this problem. The data obtained can be used in biomedical and therapeutic research, where deuterium can be considered as an adjuvant regulator of biological processes in the obesity model.

## 4. Materials and Methods

### 4.1. Physicochemical Analysis of Water with Different Deuterium Ratios

Water with different deuterium ratios was used in this study: deuterium-depleted water (DDW) with a D/H of 10 ppm (NPO “Almaz,” Tambov, Russian Federation) and MilliQ-system water (Millipore, Great Britain) with a D/H of 150 ppm, which served as a standard. MilliQ water and DDW have no differences in physical (except small weight changes) or chemical characteristics or in trace element composition, except the deuterium content, which excludes the multifactor influence in the system for all comparison groups.

The deuterium content in the water was measured by multipass laser absorption spectroscopy on an Isotopic Water Analyzer-912-0032 (Los Gatos Research, Inc., San Jose, CA, USA). Detailed description of the method was presented in previous studies [29,30].

Chemical analysis of water with different deuterium ratios was performed by inductively coupled plasma-mass spectrometry on an ICP-QMS Agilent 7500CE spectrometer (Agilent Technologies, Santa Clara, CA, USA). Detailed description of the methods was presented in previous studies [29,30]. Calibration solutions with a high range of element concentrations (from 0.1 μg∙L^−1^ to 100 μg∙L^−1^) were used to calibrate the device. The solutions were prepared based on the international standard 2.74473.0100 “ICP Multi Element Standard Solution XXI CertiPUR*^®^*”. The concentrations of the elements in the MilliQ water and DDW did not exceed the upper detection limit.

### 4.2. Animals and Housing Conditions

Detailed description of the animals and housing conditions was presented in previous study [34]. Forty adult male Wistar rats, 135 ± 10 g, were used in this study. All animal procedures were approved by the Ethical Committee of Taras Shevchenko National University of Kyiv. Animal care and manipulation was conducted in accordance with the general ethical principles of the Council of Europe Convention for the Protection of Vertebrate Animals used for Experimental and other Scientific Purposes (1997) and other international agreements and national legislation in this field.

The experiments started after 7 days of animal acclimation in the animal facility of Taras Shevchenko National University of Kyiv, maintained under constant conditions of temperature (22 ± 3 °C), humidity (60 ± 5%), and light (12 h light/12 h dark cycle). Standard rodent food and water were provided *ad libitum*.

### 4.3. Experimental Design

After acclimatization, rats were randomly divided into two experimental groups and housed in polypropylene cages at the vivarium under the conditions described above with access to water (MilliQ water with 150 ppm D/H) and their assigned chow *ad libitum*. During the experimental period, rats in the control group (*n* = 20) were fed standard laboratory chow (6.7% fat, 21% protein and 55.1% carbohydrate, 15.27 kJ·g^−1^; Laboratory Rodent Diet #5001, LabDiet). The diet-induced obesity (DIO) group (n = 20) was fed a high-fat diet (38.8% fat, 15.5% protein and 45.7% carbohydrate, 28.71 kJ·g^−1^). Normal rat chow was supplemented to create DIO food as follows: 60% standard chow, 10% pork fat, 10% eggs, 9% sucrose, 5% dry milk, 5% peanuts and 1% vegetable oil [34,52].

**Figure 4:**
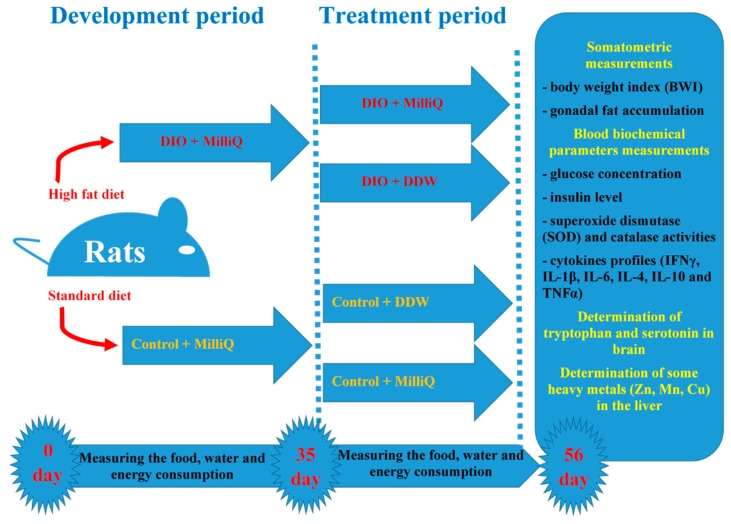
The scheme of the experimental design.

On the 35th day after the start of the experiment, half of the animals from the control and DIO groups were randomly selected and subjected to the treatment. These rats had *ad libitum* access to deuterium-depleted water (DDW) for the next 3 weeks. The other animals from each group had *ad libitum* access to MilliQ water for the same period. The scheme of the experimental design is shown in Figure 4.

Thus, there were four experimental groups with 10 animals in each: (1) Control+ MilliQ; (2) Control + DDW; (3) DIO + MilliQ; and (4) DIO + DDW.

### 4.4. Somatometric and Nutritional Assessments

Food and water consumption was measured daily at the same time (09:00 to 10:00 h). The daily energy consumption per rat (kJ/day/rat) was calculated as the average daily food intake (g) per rat multiplied by the total energy of the chow diet, which was 15.27 kJ·g^−1^ for the standard laboratory diet and 28.71 kJ·g^−1^ for the high-fat diet [52].

All animals were weighed once a week (after four hours of fasting), and at each week *i*, the percentage of weight variation Δ*w* for each animal with respect to the initial weight *w_0_* was determined as Δ*w = (w_i_ − w_0_)/w_0_·100*, where *w_i_* is the weight of the animal at week *i*.

At the scheduled death dates, the nasoanal lengths and body weight were measured, and the body weight index (BWI) of all animals was calculated. This BWI was calculated as the ratio of the body weight (g) of the rat to the square of the body length (cm^2^) [78].

At the end of the 8th week, the animals were fasted overnight and then sacrificed. The blood was collected and used for the estimation of blood glucose levels. Serum collected for the determination of other biochemical parameters was prepared by centrifugation (Eppendorf 5810, Hamburg, Germany) at 1000× *g* for 30 min of blood samples previously incubated (CB210 Binder, Tuttlingen, Germany) at 37 °C for at least 30 min. The brain of each rat was rapidly removed, weighed and separated into the left and right cerebral hemispheres. After laparotomy, gonadal fat was isolated, dried and weighed. The liver [78] was also removed for sample collection. Biological materials were stored in liquid nitrogen until used for biochemical analysis.

### 4.5. Biochemical Analysis

Blood glucose levels were evaluated using a Glucophot-II glucometer (Norma, Ukraine). The concentrations of insulin and cytokines (interferon gamma (IFNγ), interleukins (ILs) -1β, -6, -4, -10 and transforming growth factor-alpha (TNFα)) were measured in serum by enzyme-linked immunosorbent assay following standard protocols. Detailed description of the methods was presented in a previous study [34]. Samples were contained in 96-well ELISA plates (Thermo Fisher Scientific, Waltham, MA, USA), the corresponding primary antibodies (Santa Cruz Biotechnology, Inc., USA), horseradish peroxidase (Sigma-Aldrich, St. Louis, MO, USA). Plates were read at 492 nm by a microplate reader Quant^TM^ (BioTek Instruments, Inc., Winooski, VT, USA).

The activities of antioxidant enzymes, namely, superoxide dismutase (SOD) and catalase, were determined by spectrophotometric assays (all reagents were from Sigma-Aldrich, St. Louis, MO, USA). Measurements were performed on a spectrophotometer Smart SpecTMPlus (BioRad, Hercules, CA, USA). SOD activity was measured by a method [79] based on the capability of the enzyme to inhibit the autooxidation of adrenaline (epinephrine) [34]. Catalase activity was measured by a previously described method [80].

The protein concentration was determined by the Bradford method using bovine serum albumin as a standard [81].

Serotonin and tryptophan in the brain levels were measured according to a previously described method [37] (all reagents were from Sigma-Aldrich, USA). An Eppendorf 5810 centrifuge was used (Eppendorf, Hamburg, Germany). Measurements were performed on a Shimadzu RF 1501 spectrofluorophotometer (Shimadzu, Japan). The tryptophan and serotonin content in the brain was determined using ion-exchange chromatography and fluorescence, as described previously [82].

### 4.6. Heavy Metal Analysis in Rat Liver

The analysis of certain heavy metals (Zn, Cu, and Mn) in the rat liver was performed by an atomic absorption method using a C-115M1-PC spectrophotometer (SELMI, Sumy, Ukraine). Recording of the analytical signal was performed using computer-analytical complex CAS-101. The manufacturer carried out all instrument calibration procedures. Preparation of the rat liver samples for analysis was carried out according to a standard protocol [83].

### 4.7. Statistical Analysis

The biochemical estimation data were reported as the mean ± SD for each group (*n* = 10). Statistical analyses were performed using Student’s t-test in Origin 8.0 software. Differences were considered to be statistically significant at values of *p* < 0.05.

## 5. Conclusions

In summary, our findings in a rat model of obesity in vivo demonstrate that DDW can normalize metabolic parameters and markedly reduce body fat. Under low deuterium content (below natural levels in drinking water) there was a reduction in systemic inflammation caused by obesity. Our data can be used in future biomedical and therapeutic research, where deuterium can be considered as an adjuvant regulator of biological processes in the obesity model.

## Figures and Tables

**Table 1:** End of experiment biochemical parameters of control and DIO rats treated with MilliQ water or DDW. Values are expressed as the mean ± SD (*n* = 10); * *p* < 0.05 significantly different from the Control + MilliQ group; # *p* < 0.05 significantly different from the DIO + MilliQ group.

Parameters	Experimental Group			
	Control + MilliQ	Control + DDW	DIO + MilliQ	DIO + DDW
**Biochemical Parameters**				
Glucose (blood),	4.5 ± 0.4	4.2 ± 0.3	6.5 ± 0.8 *	4.9 ± 0.03
Insulin (serum), rel. un/mL of total protein	0.312 ± 0.066	0.298 ± 0.029 #	0.141 ± 0.011 *	0.120 ± 0.024 *
Catalase activities (serum), µmol Н_2_О_2_∙mg^−1^∙min^−1^	5.68 ± 0.87	7.00 ± 0.26 #	2.43 ± 0.33 *	6.18 ± 1.21 #
SOD activities (serum), c.u./mg∙min	27.44 ± 2.41	15.62 ± 3.24 *,#	4.78 ± 0.99 *	10.77 ± 2.18 *,#
Tryptophan (brain), µg∙g^−1^_tissue_	9.57 ± 1.98	8.74 ± 1.57 #	4.89 ± 0.83 *	6.89 ± 1.89 #
Serotonin (brain), µg∙g^−1^_tissue_	1.23 ± 0.27	1.06 ± 0.24	0.64 ± 0.16 *	1.16 ± 0.45
**Heavy Metals (liver)**				
Zn, µg∙g^−1^_tissue_	36.024 ± 3.822	32.205 ± 3.767 #	25.080 ± 3.128 *	35.195 ± 3.038 #
Mn, µg∙g^−1^_tissue_	2.204 ± 0.246	1.910 ± 0.137 #	1.586 ± 0.102 *	1.773 ± 0.205
Cu, µg∙g^−1^_tissue_	3.184 ± 0.265	3.059 ± 0.314	3.521 ± 0.483	3.480 ± 0.337
